# Epidemiologic trends and geographic distribution of esophageal cancer in Canada: A national population‐based study

**DOI:** 10.1002/cam4.2700

**Published:** 2019-11-12

**Authors:** Leila Cattelan, Feras M. Ghazawi, Michelle Le, François Lagacé, Evgeny Savin, Andrei Zubarev, Jennifer Gantchev, Marcel Tomaszewski, Denis Sasseville, Kevin Waschke, Ivan V. Litvinov

**Affiliations:** ^1^ Division of Dermatology Department of Medicine McGill University Montreal Quebec Canada; ^2^ Department of Medicine University of Ottawa Ottawa Ontario Canada; ^3^ Division of Gastroenterology Department of Medicine McGill University Montreal Quebec Canada

**Keywords:** Barrett's esophagus, Canada, epidemiology, esophageal adenocarcinoma, esophageal cancer, esophageal squamous cell carcinoma, gastroesophageal reflux disease (GERD), geographic clustering, great lakes, incidence, obesity, pollution, risk factors, smoking

## Abstract

**Background:**

Esophageal cancer can be subdivided into two main histological subtypes with significant variability in their etiology and epidemiology. The incidence of esophageal adenocarcinoma (AC) is increasing across the developed countries, whereas the incidence of esophageal squamous cell carcinoma (SCC) is declining. Several risk factors have been identified in the pathogenesis of each subtype, however, their epidemiologic characteristics and distribution throughout Canada remain poorly understood.

**Methods:**

We performed a retrospective analysis of demographic data across Canada from 1992 to 2010 using two independent population‐based cancer registries. The incidence of esophageal cancer, for each subtype, was examined at the levels of provinces/territories, cities, and postal codes.

**Results:**

A total of 19 790 patients were diagnosed with esophageal cancer in Canada between 1992 and 2010; 74% were males. The average national incidence rate was 33.5 cases per million individuals per year. Incidence of esophageal AC increased over time, with notable high‐incidence rates on the Vancouver Island, the coasts of the Great Lakes, and the coasts of the Northumberland Strait in the Maritimes. The overall incidence of esophageal SCC has decreased. However, high incidence of esophageal SCC was detected in the Vancouver city, rural eastern Québec, and in the Maritimes. We also report clustering for each subtype using postal codes, which sheds light onto new avenues of research for potential environmental etiologies.

**Conclusions:**

This study, for the first time, provides a detailed analysis on the burden of esophageal cancer in Canada, revealing important geographic clustering trends.

## Graphical abstract

We demonstrate clustering of esophageal adenocarcinoma in three areas of Canada (in the Maritime provinces, around the great lakes, and in British Columbia) mirroring results from a recent report in the United States. In these regions, certain lifestyle factors and environmental pollutants may play an important role in pathogenesis. Clustering of esophageal Squamous Cell Carcinoma was primarily demonstrated in British Columbia which is home to a significant community of Southeast Asian immigrants.

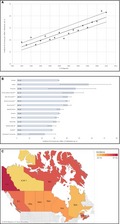

## 1 INTRODUCTION

Esophageal cancer has two main histological subtypes: squamous cell carcinoma (SCC) and adenocarcinoma (AC).^1^ Esophageal SCC is the most common of the two, representing 87% of all esophageal neoplasms in 2012.^2^ While esophageal SCC is more common in the developing countries, esophageal AC has become much more predominant across the western world.^3^ There exist many epidemiological differences between the two subtypes, however, these have not been explicitly defined on a global level until in 2014; a study by Arnold et al investigated worldwide trends of esophageal cancer incidence by histological type, reporting a global esophageal SCC incidence of 52 cases per million individuals per year, with the male predominance of 2.7 to 1.^2^ The majority (80%) of cases of esophageal SCC occurred in central and southeast Asia, with only 1.8% of cases reported in North America.^2^ The etiology of esophageal SCC is closely linked to alcohol and tobacco use.^4^ SEER data for esophageal SCC incidence in the United States examined between 1992 and 2013 demonstrated a decreasing incidence over time, from 51 cases to 26 cases per million individuals per year.^5^

Global esophageal AC affects 7 individuals per million per year with a predilection for the male gender at a ratio of 4.4:1.^2^ The majority of cases (22.8%) occurred in the northwestern Europe, with Southeast Asia and North America close behind.^2^ Within these regions, the incidence rates were much higher and favored the male gender with 8.5:1 incidence rate ratio.^2^ AC originates from Barrett's esophagus, in which normal squamous mucosa of the lower third of the esophagus undergoes dysplasia and is replaced by columnar cells resembling intestinal mucosa.^6^ The two most recognized risk factors for esophageal AC are gastroesophageal reflux disease and obesity.^7^, ^8^ While the incidence rates of several cancers have been decreasing, the incidence rate of esophageal AC has risen sixfold in the United States since 1970.^6^ Data from 13 European countries between 1983 and 1997 reported similar trends.^9^

In Canada, there have been limited studies reporting on rising rates of esophageal cancer in Ontario and in British Columbia in recent years.^10^, ^11^, ^12^ In this study, we conducted an extensive epidemiological analysis on the burden of esophageal cancer for both subtypes across all provinces and territories in Canada during the period 1992‐2010. The geographic distribution of these patients was analyzed, with the aim to better understand risk factors related to the pathogenesis of this neoplasm and to identify communities at high vs low risk for esophageal cancer.

## 2 METHODS

This study was conducted in accordance with the CISS‐RDC‐668035 and 13‐SSH‐MCG‐3749‐S001 protocols approved by the Social Sciences and Humanities Research Council of Canada (SSHRC) and the Québec Inter‐University Centre for Social Statistics (QICSS), respectively. This study was exempt from the McGill University Research Ethics Board review. The data on esophageal cancer incidence was examined using two distinct population‐based cancer databases: the Canadian Cancer Registry (CCR) and Le Registre Québécois du Cancer (LRQC) during 1992‐2010. International Classification of Disease for Oncology ICD‐O‐3 codes were used for five subtypes of esophageal AC, and three subtypes of esophageal SCC, similarly as reported in our previous studies.^13^, ^14^, ^15^, ^16^, ^17^, ^18^, ^19^, ^20^, ^21^, ^22^, ^23^, ^24^, ^25^ Due to space limitations, detailed methods are provided in the Appendix S1.

## 3 RESULTS

**Figure 1:**
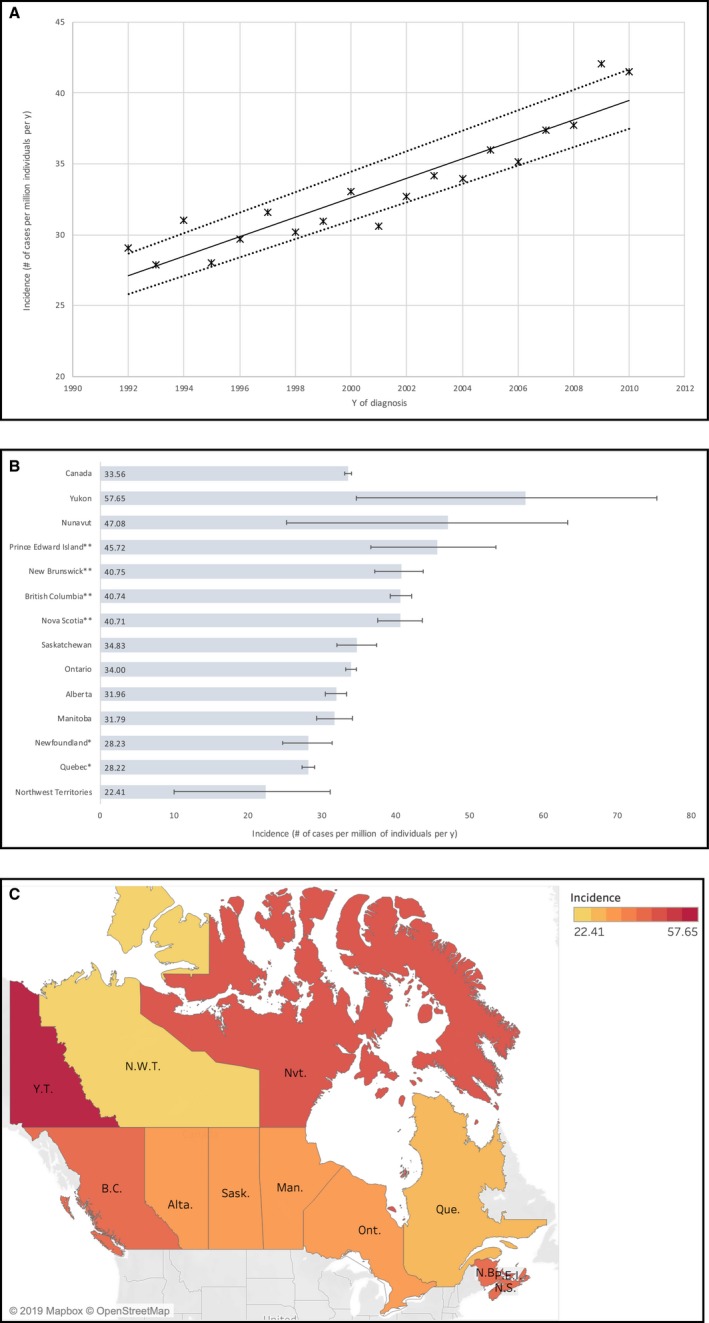
Incidence of esophageal cancer throughout Canada between 1992 and 2010 over time and by province (in cases per million individuals per year). A. Changing incidence rates for esophageal cancer between 1992 and 2010. Linear regression analysis of incidence rates over time [R2] = 0.86; *P* = .002. The slope of the line was 0.69 cases per million individuals per year. Dotted lines indicate 95% confidence interval. B. Age‐standardized incidence rates of esophageal cancer across Canadian provinces between 1992 and 2010. *Statistically significant lower incidence rates (*P* < .05) compared to the Canadian average. **Statistically significant higher incidence rates (*P* < .05) compared to the Canadian average. C. Esophageal cancer incidence trends by province in Canada. Geographic maps illustrate age‐standardized incidence rates of esophageal cancer relative to the national average based on the Canadian Cancer Registry/ Le Registre Québecois du Cancer databases

A general overview of demographic characteristics for esophageal cancer and its two histologic subtypes are presented in Table 1. In total, 19 790 patients were diagnosed with esophageal cancer. The majority (74%) were males, while 26% were females, with a male:female IRR of 2.9:1.0. The average age of diagnosis was 67.5 ± 0.8 years, with 92% of patients being > 60. The crude annual incidence of esophageal cancer showed a steady upward trend (Figure 1A); in 1992 the incidence rate was of 29.1 cases per million individuals per year; in 2010 this value had risen to 41.5, representing an increase of 43% over 19 years. The average national incidence rate for esophageal cancer between 1992 and 2010 was 33.5 cases per million individuals per year. The age‐standardized national incidence rate was 23.0 cases per year. Geographic analysis of esophageal cancer trends throughout the country is presented in Figure 1B,C. On the provincial level, Prince Edward Island (PEI), Nova Scotia (NS), New Brunswick (NB), and British Columbia (BC) had significantly higher annual age‐standardized incidence rates than that of the national average, of up to 45.7 cases per million individuals in PEI. In contrast, Newfoundland and Labrador and Quebec (QC) had significantly lower incidence rates.

**Table 1:** Epidemiologic characteristics of esophageal cancer and its two major histological subtypes between 1992 and 2010

A: Incidence by sex						
Subtype	Number of males	% of females	Number of females	% females	Average crude incidence rate in # cases per million individuals per year and (95% confidence intervals)	Average ASIR in # cases per million individuals per year and (95% confidence intervals)
Esophageal Cancer	14 635	70.01	5140	25.99	33.49 (33.02‐33.96)	23.01 (22.62‐23.40)
Esophageal AC	9005	84.36	1670	15.64	18.08 (17.74‐18.43)	12.59 (12.30‐12.89)
Esophageal SCC	5630	61.87	3470	38.13	15.41 (15.09‐15.73)	10.42 (10.16‐10.69)

B: Incidence by age group									
Subtype	<30	30‐39	40‐49	50‐59	60‐69	70‐79	80‐89	90+	Average age of diagnosis
Esophageal Cancer	0	3.03	13.42	38.89	85.68	144.51	129.13	58.88	67.52 ± 0.76
Esophageal AC	0	2.31	9.32	23.16	43.95	73.65	64.50	26.44	66.40 ± 1.01
Esophageal SCC	0	0.73	4.11	15.74	41.74	70.86	63.63	32.44	68.30 ± 1.14

Incidence rates for Canadian cities corroborated these trends and revealed certain clustering of cases throughout the country, as seen in Tables S1A,B and Figure S1. Of the 20 cities with high incidence of esophageal cancer, 10 (50%) were in Ontario (ON), 9 (45%) were in BC, and 1 (5%) was in Saskatchewan (SK). In contrast, of the 20 low‐incidence cities in Canada, 11 (55%) were located in QC, which also had a significantly lower provincial incidence rate than the rest of Canada. On further assessment, 8/11 of low‐incidence cities in QC were part of the Greater Montreal Area. We were able to observe high incidence areas on the Vancouver Island (BC), and in Southern Ontario, which encompasses Eastern, Central, and Western Ontario (Figure S1A).

Subsequently, the distribution of esophageal cancer patients was examined within cities by analyzing individual Forward Sortation Areas (FSAs). Each FSA corresponds to an area where all postal codes share the first three entries (eg, H4A). There are 1648 FSAs in Canada; 15.7% (258/1648) of these FSAs were excluded from this study as their populations were less than 5000, as per Statistics Canada regulations. High concentration of esophageal cancer cases was observed on the Vancouver Island, in Eastern Ontario, Newfoundland's Avalon Peninsula, and surrounding the Northumberland Strait on the coasts of PEI and Nova Scotia (Table S2A; Figure S1B). Several statistically‐significant zero‐incidence FSAs were also identified; areas in which zero cases of esophageal cancer had been recorded between 1992 and 2010. These 18 FSAs are presented in Table S2B, with the majority being located in Central Ontario and in the Greater Toronto Area (GTA).

### 3.1 Analysis of esophageal adenocarcinoma

**Figure 2:**
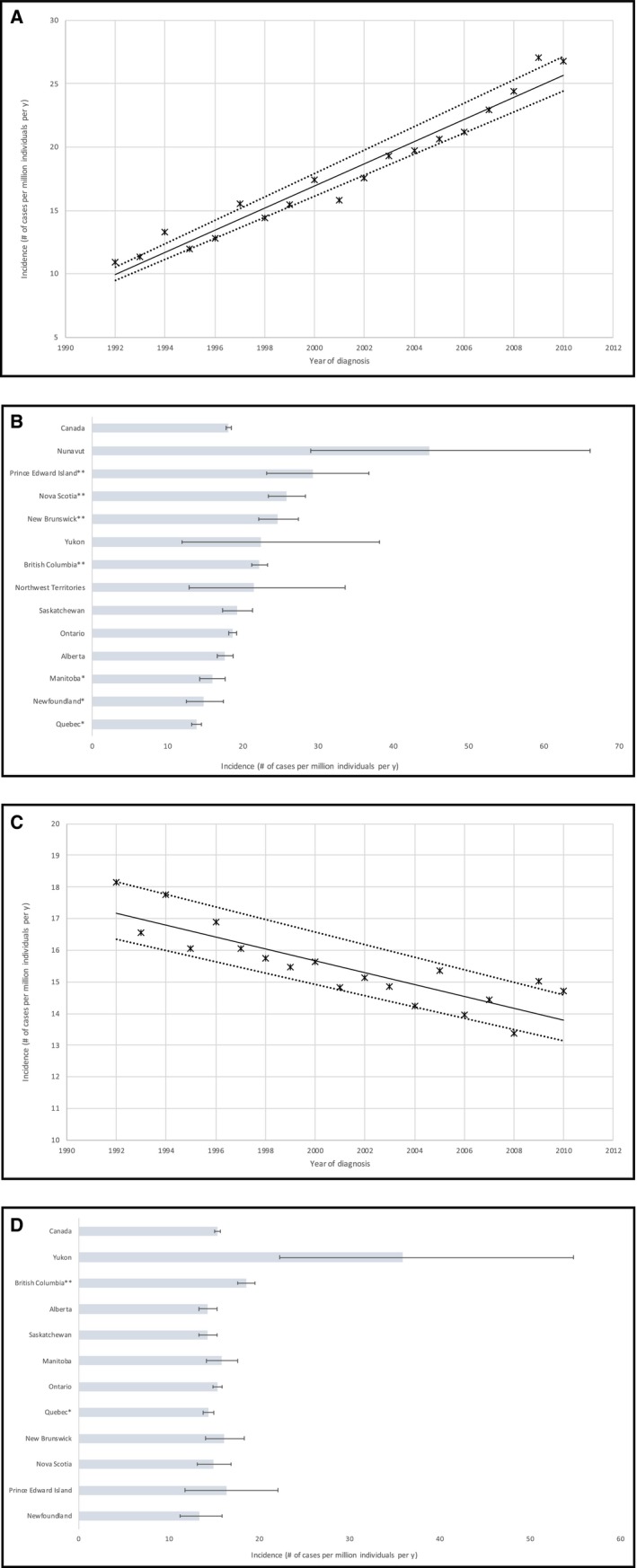
Incidence of esophageal cancer subtypes throughout Canada between 1992 and 2010 over time and by province (in cases per million individuals per year). A. Changing incidence rates for esophageal adenocarcinoma between 1992 and 2010. Linear regression analysis of incidence rates over time [R2] = 0.95; *P* = .002. The slope of the line was 0.87 cases per million individuals per year. Dotted lines indicate 95% confidence interval. B. Age‐standardized incidence rates of esophageal adenocarcinoma across Canadian provinces between 1992 and 2010. *Statistically significant lower incidence rate (*P* < .05) compared to Canadian average. **Statistically significant higher incidence rates (*P* < .05) compared to Canadian average. C. Changing incidence rates for esophageal squamous cell carcinoma between 1992 and 2010. Linear regression analysis incidence rate over time [R2] = 0.74; *P* = .002. The slope of the line was −0.19 cases per million individuals per year. Dotted lines indicate 95% confidence interval. D. Age‐standardized incidence rates of esophageal squamous cell carcinoma (per 1 million individuals per year) in the Canadian provinces between 1992 and 2010. *Statistically significant lower incidence rate (*P* < .05) compared to Canadian average. **Statistically significant higher incidence rates (*P* < .05) compared to Canadian average

Esophageal AC was diagnosed in 10 675 Canadians between 1992 and 2010, of which 84.4% of patients were male and the average age at the time of diagnosis was 66.4 ± 1.0 years (Table 1). The crude average incidence rate for esophageal AC was 18.1 cases per million individuals per year. In 1992, the incidence rate of esophageal AC was 10.9 cases per million individuals per year. By 2010 this value had risen to 26.8 cases, representing > 2‐fold increase over 19 years (Figure 2A). The age‐standardized national incidence rate was 12.6 cases per year.

**Figure 3:**
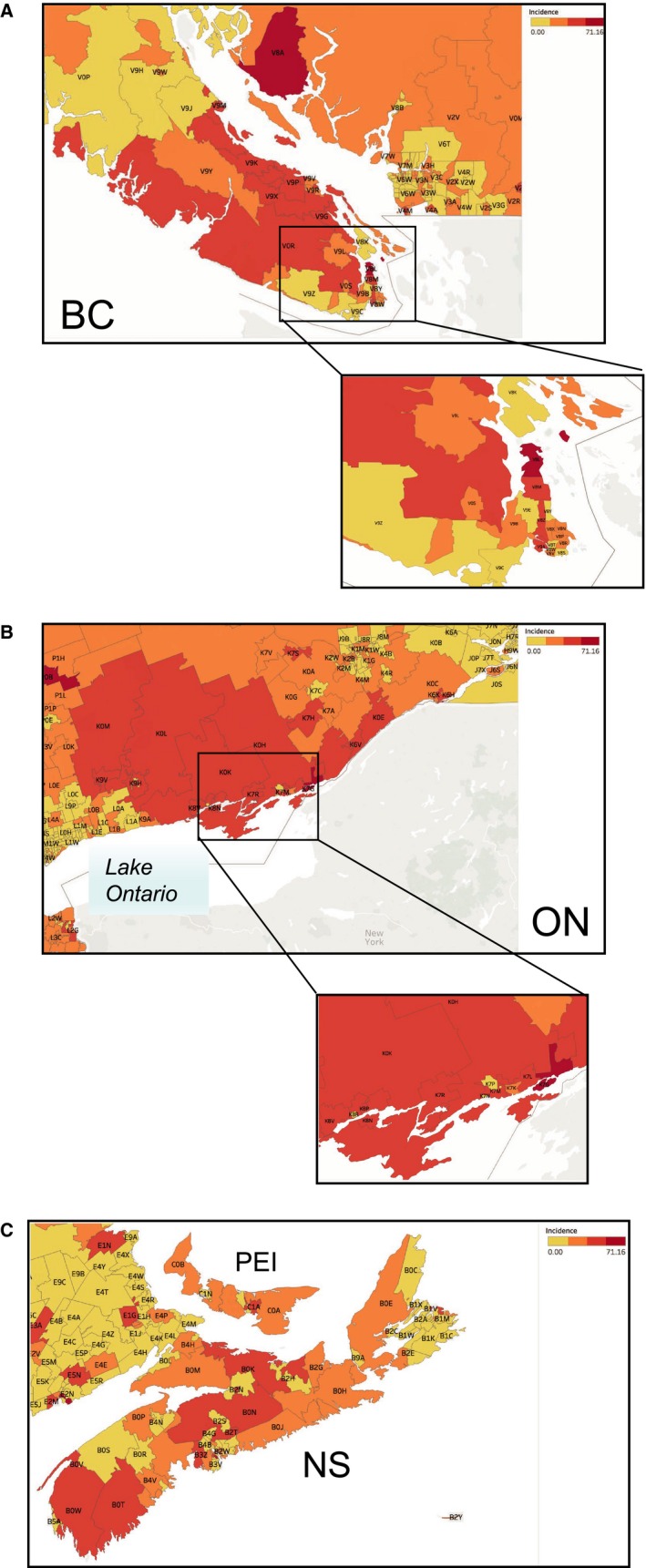
Esophageal adenocarcinoma incidence trends by FSA in Canada. Geographic maps illustrating incidence rates of esophageal adenocarcinoma (cases per million individuals per year) relative to the national average based on the Canadian Cancer Registry/ Le Registre Québécois du Cancer databases. A: Esophageal adenocarcinoma incidence trends by FSA in British Columbia (BC). This figure includes the highest incidence FSA, V8L in Sidney, North Saanich, BC. B: Esophageal adenocarcinoma incidence trends by FSA in Ontario. This figure shows high incidence esophageal adenocarcinoma FSAs in Eastern Ontario surrounding Lake Ontario. C: Esophageal adenocarcinoma incidence trends by FSA in the Maritimes. This figure shows high incidence esophageal adenocarcinoma FSAs in Nova Scotia and PEI

On the provincial level, PEI, NS, NB, and BC had significantly higher incidence rates than the national average, of up to 29.4 cases per million individuals per year in the PEI (Figure 2B). In contrast, MB, NF, and QC had significantly lower incidence rates. Incidence rates for Canadian cities corroborated these trends and revealed patient clusters (Tables S3A,B). The distribution of esophageal AC patients within cities by FSA is presented in Table 2, and Figure 3. A notably high incidence was found for the FSA V8L, representing a borough within the city of Sidney, in North Saanich on the Vancouver Island, with the rate of 71.2 cases per million individuals per year, fourfold higher than the national average. The city of Sidney was also recorded as the city with the highest incidence of esophageal AC in Canada (95.4 cases per million individuals per year). Three clusters of esophageal AC cases were found, in the west coast of BC, rural eastern ON, and in the Maritimes surrounding Northumberland Strait. These results are further discussed in Appendix S2.

**Table 2:** List of Populous Forward Sortation Areas (FSA) in Canada with high crude incidence rates of esophageal adenocarcinoma from 1992 to 2010. All population numbers are rounded to the nearest thousand

FSA	Province	Cases	Average Population	Crude incidence per million individuals per year	Lower 95% CI	Higher 95% CI
B0E	NS	20	30 000	34.97	21.36	54.01
B0K	NS	30	38 000	41.13	27.75	58.71
B0N	NS	30	44 000	36.15	24.39	51.60
B0T	NS	15	17 000	47.73	26.71	78.73
B0W	NS	25	33 000	39.49	25.56	58.29
B1H	NS	10	11 000	48.78	23.39	89.70
B2N	NS	20	29 000	36.45	22.26	56.29
B2T	NS	10	14 000	38.99	18.70	71.70
B3A	NS	15	20 000	38.62	21.62	63.70
B3N	NS	10	12 000	43.57	20.89	80.13
B3P	NS	10	8000	62.36	29.90	114.68
B3Z	NS	10	11 000	48.96	23.48	90.04
C0A	PEI	25	43 000	30.32	19.62	44.77
C1A	PEI	25	31 000	42.17	27.29	62.26
E1N	NB	10	11 000	47.54	22.80	87.44
E2J	NB	20	16 000	66.71	40.75	103.02
E2M	NB	15	19 000	41.27	23.10	68.07
E3A	NB	20	27 000	38.40	23.46	59.31
E3V	NB	10	12 000	42.17	20.22	77.56
E5N	NB	10	11 000	46.70	22.39	85.88
E7M	NB	10	10 000	54.26	26.02	99.78
G1H	QC	20	29 000	36.41	22.24	56.23
G1M	QC	15	18 000	44.45	24.88	73.32
G6G	QC	15	20 000	38.66	21.64	63.77
J1H	QC	20	29 000	36.88	22.53	56.96
J6S	QC	15	23 000	34.58	19.35	57.04
K0A	ON (Eastern)	50	91 000	28.76	21.35	37.92
K0C	ON (Eastern)	30	51 000	30.92	20.86	44.14
K0E	ON (Eastern)	35	39 000	47.83	33.32	66.53
K0G	ON (Eastern)	20	33 000	31.51	19.24	48.66
K0H	ON (Eastern)	30	42 000	37.49	25.29	53.51
K0K	ON (Eastern)	75	103 000	38.23	30.07	47.93
K0L	ON (Eastern)	50	68 000	38.52	28.59	50.78
K0M	ON (Eastern)	40	46 000	45.39	32.43	61.81
K2A	ON (Eastern)	15	15 000	51.16	28.64	84.39
K6H	ON (Eastern)	25	29 000	44.75	28.96	66.07
K6V	ON (Eastern)	25	28 000	46.33	29.98	68.39
K7G	ON (Eastern)	10	8000	63.03	30.23	115.92
K7K	ON (Eastern)	20	31 000	34.46	21.05	53.21
K7L	ON (Eastern)	15	21 000	38.12	21.34	62.87
K7M	ON (Eastern)	35	45 000	40.76	28.39	56.69
K7S	ON (Eastern)	10	12 000	44.57	21.37	81.96
K8N	ON (Eastern)	20	26 000	39.89	24.36	61.60
K8P	ON (Eastern)	15	20 000	38.53	21.56	63.55
K8V	ON (Eastern)	20	27 000	39.17	23.93	60.50
K9H	ON (Eastern)	25	26 000	50.47	32.66	74.51
K9J	ON (Eastern)	35	45 000	41.18	28.69	57.28
K9V	ON (Eastern)	20	25 000	42.41	25.91	65.50
L0K	ON (Central)	20	33 000	32.19	19.66	49.72
L0P	ON (Central)	10	11 000	47.76	22.90	87.83
L2E	ON (Central)	15	21 000	36.87	20.64	60.82
L2N	ON (Central)	20	32 000	33.35	20.37	51.51
L2P	ON (Central)	10	13 000	39.04	18.72	71.80
L3V	ON (Central)	25	41 000	32.01	20.71	47.25
L7N	ON (Central)	15	13 000	61.29	34.31	101.10
L8L	ON (Central)	20	34 000	31.40	19.18	48.50
L8P	ON (Central)	15	22 000	35.79	20.03	59.03
L8T	ON (Central)	15	19 000	40.97	22.93	67.57
L8V	ON (Central)	15	22 000	36.69	20.53	60.51
L9A	ON (Central)	15	23 000	33.71	18.87	55.60
L9H	ON (Central)	25	31 000	42.44	27.47	62.66
N0A	ON (Western)	20	30 000	35.56	18.47	42.14
N0H	ON (Western)	25	45 000	29.32	21.72	54.92
N1A	ON (Western)	10	12 000	44.19	18.97	43.28
N2H	ON (Western)	15	21 000	37.45	20.96	61.77
N3B	ON (Western)	10	10 000	50.85	24.39	93.52
N3Y	ON (Western)	15	22 000	36.03	20.17	59.43
N4N	ON (Western)	10	9000	56.35	27.02	103.63
N4W	ON (Western)	10	10 000	54.15	25.97	99.58
N5C	ON (Western)	10	14 000	38.96	18.68	71.64
N7A	ON (Western)	10	12 000	45.06	21.61	82.87
N7T	ON (Western)	20	27 000	38.91	23.77	60.10
N8S	ON (Western)	15	23 000	34.49	19.30	56.89
P0B	ON (Northern)	10	8000	62.51	29.97	114.95
P1B	ON (Northern)	20	34 000	30.72	18.77	47.45
P2N	ON (Northern)	10	8000	65.87	31.59	121.14
P4N	ON (Northern)	20	28 000	37.04	22.62	57.20
P5A	ON (Northern)	10	12 000	43.39	20.81	79.80
P6B	ON (Northern)	15	24 000	33.42	18.71	55.13
P7B	ON (Northern)	25	30 000	43.67	28.26	64.47
P7C	ON (Northern)	25	29 000	45.23	29.27	66.77
P7E	ON (Northern)	15	22 000	35.79	20.03	59.03
R2Y	MB	15	20 000	39.28	21.98	64.78
S0E	SK	20	34 000	30.56	18.67	47.20
S0L	SK	20	32 000	32.82	20.05	50.69
S4P	SK	10	12 000	42.17	20.22	77.56
S6H	SK	25	30 000	44.20	28.60	65.25
T2E	AB	20	32 000	32.94	20.12	50.87
V0E	BC	45	65 000	36.70	26.77	49.10
V0H	BC	35	51 000	36.13	25.17	50.25
V0R	BC	45	64 000	36.92	26.93	49.40
V0X	BC	15	20 000	39.55	22.14	65.24
V1C	BC	15	24 000	33.28	18.63	54.90
V1J	BC	15	22 000	36.30	20.32	59.87
V1T	BC	20	31 000	33.91	20.71	52.37
V1Y	BC	20	32 000	32.93	20.11	50.85
V2A	BC	25	35 000	37.55	24.30	55.43
V2B	BC	30	36 000	44.23	29.84	63.14
V2P	BC	25	33 000	39.29	25.43	58.00
V2S	BC	25	44 000	29.59	19.15	43.69
V3A	BC	25	40 000	32.79	21.22	48.40
V3L	BC	15	23 000	34.61	19.37	57.09
V4A	BC	20	35 000	30.19	18.44	46.62
V4K	BC	15	24 000	33.42	18.71	55.13
V4P	BC	10	12 000	45.65	21.89	83.95
V4T	BC	15	21 000	37.92	21.22	62.54
V8A	BC	20	18 000	58.71	35.86	90.67
V8L	BC	30	22 000	71.16	48.01	101.58
V8R	BC	15	23 000	34.78	19.47	57.36
V8V	BC	15	24 000	33.21	18.59	54.78
V8X	BC	15	22 000	35.21	19.71	58.08
V8Z	BC	25	27 000	48.46	31.36	71.54
V9A	BC	25	35 000	37.85	24.50	55.88
V9B	BC	20	32 000	32.56	19.89	50.28
V9G	BC	10	10 000	50.56	24.24	92.98
V9K	BC	10	14 000	38.90	18.65	71.54
V9N	BC	20	27 000	38.35	23.42	59.22
V9P	BC	20	21 000	51.35	31.36	79.30
V9S	BC	15	16 000	50.87	28.47	83.90

### 3.2 Analysis of esophageal squamous cell carcinoma

**Figure 4:**
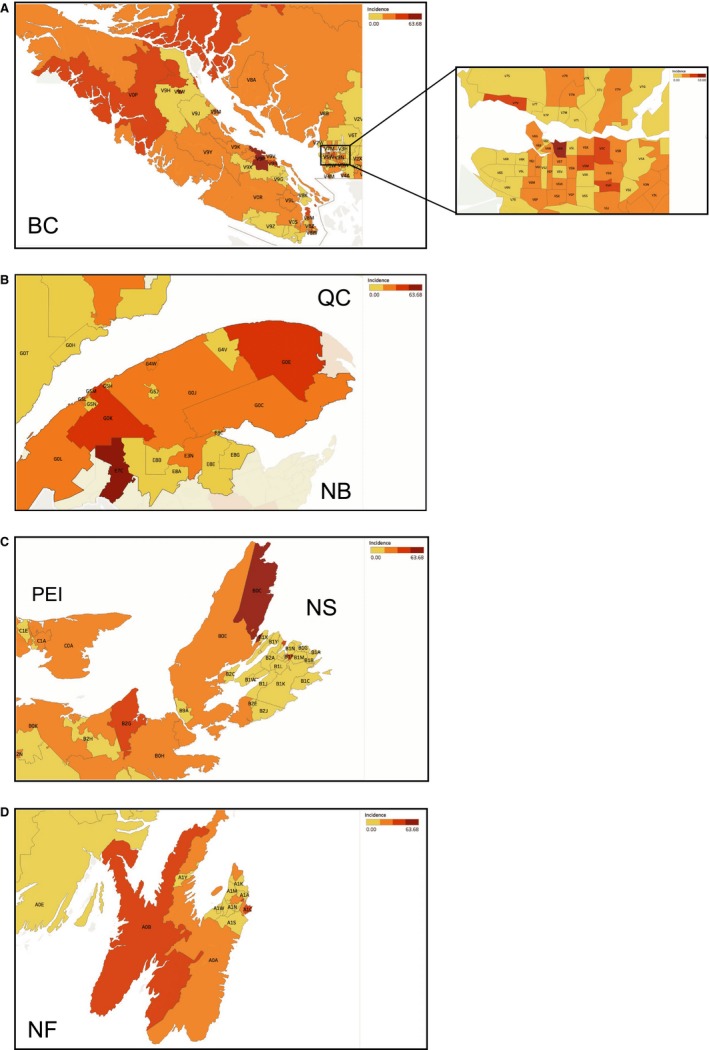
Esophageal squamous cell carcinoma incidence trends by FSA in Canada. Geographic maps illustrating incidence rates of esophageal squamous cell carcinoma (cases per million individuals per year) relative to the national average based on the Canadian Cancer Registry/Le Registre Québecois du Cancer databases. A, High incidence esophageal squamous cell carcinoma FSAs in British Columbia. This figure includes the highest incidence FSA, V6A in Vancouver, BC. B, High incidence esophageal squamous cell carcinoma FSAs in Quebec and New Brunswick. C, High incidence esophageal squamous cell carcinoma FSAs in Nova Scotia and PEI. D, High incidence esophageal squamous cell carcinoma FSAs in Newfoundland and Labrador province

Esophageal SCC was diagnosed in 9115 Canadians between 1992 and 2010, with 61.9% male patients, and an average age at diagnosis of 68.3 ± 1.1 years (Table 1). The crude average incidence rate for esophageal SCC was 15.4 cases per million individuals per year. In 1992, the incidence rate was 18.2 cases per million individuals per year. In 2010, this value had decreased to 14.7 cases, representing a 19% decrease over 19 years (Figure 2C). The age‐standardized national incidence rate was 10.4 cases per year. On the provincial level (Figure 2D), only BC exhibited significantly higher incidence rates than the national average (18.6 cases per million individuals per year). In contrast, QC had a significantly lower incidence rate of 14.4 cases per million individuals per year. Analysis by FSA (Table 3 and Figure 4A) showed a notable high‐incidence in V6A postal code, a borough within the Vancouver city, encompassing Strathcona, Chinatown and Downtown Eastside (DTES) with a rate of 63.7 cases per million individuals per year, approximately four times higher than the national average. Significant clusters of esophageal SCC cases were found in the west coast of BC, rural QC and NB, and in the Maritimes. Results at the city and FSA levels are further discussed in Appendix S2 and can be viewed in Figure 4A‐D.

**Table 3:** List of Populous Forward Sortation Areas (FSA) in Canada with high crude incidence rates of esophageal squamous cell carcinoma from 1992 to 2010

FSA	Province	Cases	Average population	Crude incidence per million individuals per year	Lower 95% CI	Higher 95% CI
A0B	NF	15	23 610	33.44	18.70	55.15
A1C	NF	10	14 280	36.86	17.64	67.79
B0C	NS	5	5300	49.65	16.00	115.87
B0K	NS	20	38 390	27.42	16.74	42.35
B1P	NS	10	16 060	32.77	15.69	60.27
B1V	NS	5	7020	37.49	12.08	87.48
B2G	NS	10	14 080	37.38	17.90	68.75
E3A	NB	15	27 410	28.80	16.11	47.51
E7C	NB	5	5500	47.85	15.42	111.66
G0E	QC	10	11 790	44.64	21.37	82.10
G0K	QC	10	13 200	39.87	19.09	73.33
G1J	QC	20	22 810	46.15	28.18	71.28
G1K	QC	15	18 270	43.21	24.17	71.28
G1L	QC	15	22 920	34.44	19.26	56.82
G1R	QC	15	16 710	47.25	26.42	77.93
H1H	QC	20	34 700	30.34	18.52	46.85
H1M	QC	15	27 510	28.70	16.05	47.34
H2H	QC	10	13 920	37.81	18.10	69.54
H2K	QC	15	27 080	29.15	16.30	48.09
H4H	QC	15	19 480	40.53	22.67	66.85
H8N	QC	15	26 490	29.80	16.67	49.16
H8P	QC	15	22 410	35.23	19.70	58.11
H9S	QC	15	21 540	36.65	20.50	60.46
J2K	QC	10	14 570	36.12	17.29	66.44
J7Z	QC	15	28 270	27.93	15.62	46.06
J8X	QC	10	10 900	48.29	23.12	88.81
K0J	ON (Eastern)	20	33 120	31.78	19.41	49.09
K2A	ON (Eastern)	15	15 430	51.16	28.62	84.39
K2B	ON (Eastern)	20	33 050	31.85	19.45	49.19
K2C	ON (Eastern)	15	27 040	29.20	16.33	48.16
K2P	ON (Eastern)	10	14 810	35.54	17.01	65.36
K6V	ON (Eastern)	20	28 400	37.06	22.63	57.25
K7A	ON (Eastern)	10	16 640	31.63	15.14	58.17
K7C	ON (Eastern)	10	15 160	34.72	16.62	63.85
K7H	ON (Eastern)	10	14 520	36.25	17.35	66.67
K8N	ON (Eastern)	15	26 390	29.92	16.73	49.34
K9J	ON (Eastern)	30	44 730	35.30	23.81	50.39
L2A	ON (Central)	10	16 200	32.49	15.55	59.75
L4Y	ON (Central)	15	24 090	32.77	18.33	54.06
L6K	ON (Central)	10	12 650	41.61	19.92	76.52
L6L	ON (Central)	15	26 320	30.00	16.78	49.48
L6T	ON (Central)	20	38 980	27.00	16.49	41.71
L7L	ON (Central)	20	36 720	28.67	17.50	44.28
L7N	ON (Central)	10	12 880	40.86	19.56	75.15
M4G	ON (Toronto)	10	16 260	32.37	15.50	59.53
M4V	ON (Toronto)	10	16 380	32.13	15.38	59.10
M6N	ON (Toronto)	25	41 110	32.01	20.71	47.25
M9N	ON (Toronto)	15	23 880	33.06	18.49	54.53
N0M	ON (Western)	30	64 370	24.53	16.55	35.02
N2G	ON (Western)	10	13 490	39.02	18.68	71.76
N3T	ON (Western)	15	26 310	30.01	16.78	49.49
N5A	ON (Western)	20	30 420	34.60	21.13	53.44
N6A	ON (Western)	10	10 850	48.51	23.22	89.21
N6B	ON (Western)	10	9970	52.79	25.27	97.09
N7A	ON (Western)	10	11 680	45.06	21.57	82.88
N8X	ON (Western)	10	16 280	32.33	15.48	59.46
N9A	ON (Western)	20	27 120	38.81	23.70	59.95
P0H	ON (Northern)	20	34 630	30.40	18.56	46.95
P0K	ON (Northern)	10	12 270	42.89	20.54	78.89
P0L	ON (Northern)	15	25 130	31.42	17.57	51.82
P1H	ON (Northern)	10	14 700	35.80	17.14	65.85
P2A	ON (Northern)	10	12 270	42.89	20.54	78.89
P3B	ON (Northern)	10	15 010	35.06	16.79	64.49
P4N	ON (Northern)	15	28 420	27.78	15.54	45.82
P5A	ON (Northern)	10	12 130	43.39	20.77	79.80
P6A	ON (Northern)	20	35 850	29.36	17.93	45.35
P6B	ON (Northern)	20	23 620	44.57	27.21	68.83
R2L	MB	10	14 680	35.85	17.16	65.94
R2W	MB	20	27 960	37.65	22.99	58.15
R3J	MB	15	27 220	29.00	16.22	47.84
R7N	MB	10	10 090	52.16	24.97	95.93
S4P	SK	10	12 480	42.17	20.19	77.56
S6H	SK	25	29 770	44.20	28.59	65.25
T1A	AB	20	26 160	40.24	24.57	62.15
T5B	AB	10	15 890	33.12	15.86	60.92
T6A	AB	10	14 710	35.78	17.13	65.80
T6E	AB	15	21 600	36.55	20.44	60.29
V0H	BC	25	50 980	25.81	16.70	38.10
V0R	BC	35	64 150	28.72	20.00	39.94
V0X	BC	15	19 960	39.55	22.12	65.24
V1R	BC	10	9950	52.90	25.32	97.28
V1Y	BC	25	31 970	41.16	26.63	60.76
V2P	BC	20	33 490	31.43	19.19	48.55
V3M	BC	20	34 410	30.59	18.68	47.25
V5C	BC	15	23 470	33.64	18.81	55.48
V5H	BC	20	32 850	32.04	19.56	49.49
V5M	BC	15	21 790	36.23	20.26	59.76
V6A	BC	20	16 530	63.68	38.88	98.35
V7V	BC	10	15 000	35.09	16.80	64.53
V8L	BC	15	22 190	35.58	19.90	58.68
V8R	BC	15	22 700	34.78	19.45	57.37
V8X	BC	15	22 420	35.21	19.69	58.08
V9A	BC	20	34 760	30.28	18.49	46.77
V9N	BC	15	27 450	28.76	16.09	47.44
V9P	BC	20	20 500	51.35	31.35	79.31
V9R	BC	20	25 050	42.02	25.66	64.90
V9S	BC	10	15 520	33.91	16.24	62.37
V9W	BC	20	27 620	38.11	23.27	58.86

## 4 DISCUSSION

To our best knowledge, no previous study performed has assessed the distribution of esophageal AC or SCC across the entire country and to this level of detail. In this work, we present important national trends corroborating those seen in similar studies from the United States, Europe, and Australia. At the national level, we report a steadily increasing overall burden of esophageal cancer in Canada, with a male predominance. Over 90% of these patients were > 60, reflecting a correlation between Canada's aging population and an increase in the current and projected incidence of cancer across the country.^26^ The average incidence rate was found to be 33.5 cases per million individuals per year, and the average age‐adjusted (to the World Standard Population) incidence rate was 23.0 cases per million individuals per year. This is consistent with worldwide trends:

Wong et al^27^ reported national age‐standardized incidence rates, standardized to the World Standard Population (in cases per million individuals per year) of 27 in Europe, 29 in North America, 34 in South America, and 29 in Australia. The authors additionally report an average male to female incidence ratio of 3:1,^27^ similar to our findings of 2.9:1.

Regarding the specific subtypes analyzed, we confirmed an important increase in esophageal AC incidence over time that has been seen in many developed countries. Age‐standardized esophageal AC incidence rates were reported by Thrift et al^3^ (in cases per million individuals per year) as 24.5 cases in Australia, 24.5 cases in the United States, and 13.9 cases in Sweden between 1999 and 2003. The proportion of obese Canadians has doubled between the 1980s and 2004.^28^ This potentially links esophageal AC to the higher rates of obesity seen in countries of a higher socioeconomic status.^2^, ^3^, ^6^, ^29^ With an aging population, patients may experience a longer duration of Gastroesophageal reflux disease (GERD) which would increase the risk of Barrett's esophagus and esophageal AC.^30^, ^31^ In addition, this increasing rate of esophageal AC may reflect the failure of screening appropriate patients or inadequate surveillance since Barrett's esophagus, GERD and obesity are potentially modifiable risk factors.^32^, ^33^

In the present study, we also described a decrease in esophageal SCC. The age‐standardized esophageal SCC incidence rates reported by Thrift et al^3^ (in cases per million individuals per year) corroborate our results: 25.1 in Australia, 18.1 in the United States, 14.0 in the Caucasian population of the United States, and 17.2 per in Sweden between 1999 and 2003. A study by Otterstatter et al^29^ reports an incidence of esophageal SCC in Canada of 13.5 cases per million individuals per year in 2004‐2006, reflecting the decreased incidence of esophageal SCC in recent years.^29^ A decline in tobacco smoking among Canadians may in part explain the decline in esophageal SCC incidence.^34^ SEER data during the period 1998‐2003 also corroborates these results; an overall incidence during that time was documented to be 18 cases per million individuals per year, slightly higher than the Canadian average.^35^ Esophageal SCC incidence in Europe tends to be much higher than in North America, likely due to much higher rates of cigarette consumption.

Geographic analysis of esophageal cancer trends throughout the country demonstrated that this malignancy localized more strongly to the Maritimes and BC. The present findings demonstrated clustering of esophageal AC cases in three particular areas of Canada; Vancouver Island, the coasts of the Great Lakes, and the coasts of the Northumberland Strait between NS and PEI. A recent study^36^ reported on clustering of esophageal cancer mortality in Caucasian men across the United States, which showed a high mortality from this cancer in the northeastern part of the country, particularly along the coasts of Lakes Michigan, Erie, and Ontario.^36^ Further analysis demonstrated high association of these areas with cigarette use, binge alcohol drinking habit, and obesity.^36^ This corroborates SEER data which reports that the highest incidence of esophageal AC is found in the Northeast and Midwest of the United States.^35^ In addition, although the heritable nature of esophageal cancer is not as strong as in some other cancers, familial clustering in high incidence areas has been observed in both esophageal cancer subtypes in previous studies.^37^, ^38^

The data in the present study demonstrate high incidence around several coastal cities of the Great Lakes, with the largest cluster surrounding Lake Ontario. The Great Lakes represent 80% of the fresh water supply in North America, providing drinking water for millions of people.^39^ A study by the Great Lakes Environmental Assessment and Mapping Project identified environmental stressors impacting this region, which may in part explain these findings.^40^ Their investigations revealed that Lakes Ontario, Erie, and Michigan scored highest on the cumulative stress index (CSI), due to being more populated and developed.^40^ Most common “stressors” ranged from toxins such as polychlorinated biphenyls and mercury, to pollution related to high shipping activity and charter, to land run‐off such as phosphorous and nitrogen.^40^ According to the World Wildlife Foundation's national assessment of Canada's freshwater, Vancouver Island, and parts of the NS and PEI were also highly affected by increased pollution related to industrial waste, urban runoff, and pipeline incidents.^41^ This accentuates the need to consider environmental exposures as an additional cardinal factor in the pathogenesis of this cancer among Canadians in these industrialized coastal regions.

Analysis of esophageal SCC trends, on the other hand, revealed high incidence in BC, rural eastern QC, and in the Maritimes. A recent report on tobacco use in Canada highlighted the overall decrease in smoking prevalence in the country, which may have contributed to the decrease of incidence of esophageal SCC.^34^, ^42^ The authors reported higher rates of cigarette consumption in NB, PEI, BC, AB, QC, and NL; these provinces happened to show higher incidence of esophageal SCC in the present study.^42^ Arnold et al identified a disproportionately high rate of esophageal SCC in central and southeast Asia.^2^ Similarly, BC has a large Southeast Asian population that may contribute to the higher incidence of esophageal SCC in that province.^43^

The highest incidence FSA corresponded to downtown Vancouver, specifically the DTES, colloquially referred to as Canada's “poorest postal code.”^44^ This low‐income area is known for unusually high rates of unemployment, crime, substance abuse, prostitution, and high incidence of infection with HIV/AIDS and hepatitis C virus.^44^ Efforts have recently been placed on providing better access to screening for certain cancers to which the DTES residents appear very vulnerable such as oral and cervical cancer.^44^, ^45^, ^46^ The possibility of high alcohol and tobacco use in this population, coupled with a lower access to education and healthcare, may make the residents of the DTES particularly vulnerable to esophageal SCC.

This study is not without limitations; in large retrospective studies using databases as those presently employed, there exists a risk of data omission or misclassification. While many other worldwide studies have noted the prevalence of esophageal adenocarcinoma to differ by ethnicity, data concerning ethnic background of Canadian patients was not collected by the CCR and LRQC databases and, hence, was not available for analysis.

It is also important to highlight that as Canada's healthcare system is a single‐tier (payer), which is funded and operated by the government, the data are collected with consistency, where each provincial and territorial cancer registry identifies tumors in its population by combining information from sources such as: cancer clinic files, radiotherapy and hematology reports, records from in‐patient hospital stays, out‐patient clinics, pathology and other laboratory/autopsy reports, radiology and screening program reports, medical billing and hospital discharge administrative databases. The CCR/LRQC performs multiple rigorous processes to ensure accuracy including an internal record linkage to identify possible duplicate records. These measures allow for high rates of detection and diagnostic accuracy of incidence data recorded by the registries.

Indeed, several studies investigated the detection rates and accuracy of diagnostic data in the largest provincial branch of the Canadian Cancer Registry: the Ontario Cancer Registry (OCR) which collects data from the most populous province. In fact, a case ascertainment of ~99%, a detection rate (detecting and accurately assigning index tumor site) of 81.4%‐96%, and a confirmation rate (correctly assigning tumor site) of 90.9% were documents by several studies,^47^, ^48^, ^49^ which confirms a high quality of data and detection rates in the examined registries.”

In conclusion, this epidemiologic study highlights areas of clustering of esophageal AC and SCC throughout Canada and provides an overview of known and potential risk factors to consider. Future analyses may confirm the existence of putative environmental risk factors presented in the present study. This report also provides the basis for locating and studying the areas of Canada that may benefit from more efforts for education and screening, as well as more focused distribution of healthcare resources in order to decrease incidence, morbidity, and mortality relating to esophageal cancer throughout the country.

## CONFLICT OF INTEREST

Authors declare they have no conflict of interest regarding the content of this article.

## AUTHOR CONTRIBUTIONS

Leila Cattelan collected and analyzed data and wrote the article. Feras M. Ghazawi analyzed data and co‐wrote the article. Michelle Le analyzed data. François Lagacé performed statistical analyses. Evgeny Savin performed statistical analyses. Andrei Zubarev performed statistical analyses. Jennifer Gantchev co‐wrote the paper. Marcel Tomaszewski co‐wrote the paper. Denis Sasseville collected data, designed and supervised the study, and co‐wrote the article. Kevin Waschke collected data, designed and supervised the study, and co‐wrote the article. Ivan V. Litvinov collected and analyzed the data, designed and supervised the study, and co‐wrote the article.

## Data Availability

All original data is publicly available through Canadian Cancer Registry, Quebec Cancer Registry and Canadian Vital Statistics databases from Statistics Canada.
