# Supplementary material for: Epidemiologic trends and geographic distribution of esophageal cancer in Canada: A national population‐based study
Source: Cancer Med. 2019 Nov 12;9(1):401–17. doi: 10.1002/cam4.2700 (PMC6943153; doi:10.1002/cam4.2700)
Supplement: Supplementary file 1 [file CAM4-9-401-s001.pdf]

## Appendix 1

### Methods (continued)

The CCR is a database of Canadian residents from 12 provinces and territories (excluding Québec), who have been diagnosed with primary tumors from 1992 to 2013, regardless of their mortality status. Data for Québec patients were obtained from the LRQC, which was only available between 1992 and 2010. Since the data from the LRQC database for Québec was only available up to 2010, we chose to limit analysis for this study between 1992 and 2010 in order to represent all Canadian provinces and territories equally. The CCR/LRQC databases provide demographic, geographic and clinical information on all afflicted with cancer Canadian patients (population of Canada was ~34 million in 2010). The variables studied were: gender, year of diagnosis, age at time of diagnosis, province, city, and postal code of residence of patients. The CCR/LRQC databases do not record data concerning certain other demographic characteristics of the patients, such as ethnic background. Cases of esophageal adenocarcinoma were defined based on the International Classification of Disease for Oncology, Third Editions (ICD-0-3) as the following: Adenocarcinoma (8140), Adenoid cystic carcinoma (8200), Adenosquamous carcinoma (8560), Mucopeidermoid carcinoma (8430), and Undifferentiated carcinoma (8020). Cases of esophageal squamous cell carcinoma were defined as: Squamous cell carcinoma (8070), Verrucous carcinoma (8051), and Basaloid squamous cell carcinoma (8083).

For incidence analyses, data on population counts nationally, by province, by city and by FSA (Forward Sortation Area) were obtained from Statistics Canada's Canadian Census of Population for 1996, 2001, 2006 and 2011 years. In Canada, postal codes consist of letters and numbers (*e.g.* H3G 1A4), where the first 3 entries, Forward Sortation Area (FSA), define a region in the country, with more than 1,600 FSAs across Canada. Prior to being released from the three

databases employed, the data must be vetted in order to verify compliance with the variety of confidentiality regulations. SSHRC/Statistics Canada requires that each cell count be rounded to a multiple of 5 using a random rounding system. In addition, no counts ≥1 and 5< of cases can be released. Therefore, we were able to delineate high incidence communities, as well as locate areas in the country, where zero cases were documented between 1992 and 2010, designated as low incidence areas.

Incidence rates and 95% confidence intervals (CI) were calculated and reported overall, by year of diagnosis and specific geographic regions that were identified by the mapping analysis. CIs were based on exact Poisson tests. Statistical significance was defined by 95% CI not overlapping with that of the national average 95% CI. The national age-standardized incidence rates for esophageal cancer and its subtypes were calculating using the WHO population standard[1]. The age-standardized incidence rates by province and by FSA for esophageal cancer and its subtypes were calculated using the Canadian population standard between 1992 and 2010. Incidence rates were plotted, and linear regression models were used to assess trends over time. Geographic maps of Canada, indicating the place of residence of patients recorded by the CCR and LRQC databases, were generated using geographic information systems software (Tableau 10.3 from Tableau Software, Seattle Washington, USA). Only FSAs with populations of at least 5,000 individuals based on census data were selected in order to reduce the risk of false-positive hits which could inflate the incidence rate.

## Appendix 2

## Results (continued)

*Analysis of esophageal adenocarcinoma*

Of the 53 cities with high incidence of esophageal AC in Canada (**Supporting Table 3A**), 15 (28%) were found in BC, 6 (11%) in NS, 2 (0.04%) in NB and 1 (0.02%) in PEI. Most importantly, 26 (49%) of these high incidence cities were located in Ontario. On closer inspection of these cities, 20 (77%) were located on the coasts of the four Great Lakes: Superior, Huron, Eerie and Ontario. On the other hand, of the 37 low-incidence cities in Canada (**Supporting Table 3B**), 20 (54%) were located in Quebec, which also had a significantly lower provincial incidence than the national average.

A notable cluster of esophageal AC cases is shown covering a significant portion of British Columbia's west coast (**Figure 3**), particularly Central and South Vancouver Island (V0R), Qualicum Beach (V9K), Parksville (V9P), Nanaimo (V9X), Central Saanich (V8M), Powell River (V8A) and Victoria (V9A and V8Z). Another cluster was identified in rural Eastern Ontario (**Figure 4A**). This included areas surrounding the shore of Lake Ontario (K0E, K0H, K0K, K0L, K0M,), Trenton (K8V), Belleville (K8N), Napanee (K7R), Perth (K7H), Brockville (K6V), Kingston (K7M, K7L) and Gananoque (K7G). The third significant cluster identified was in the Maritimes, on either side of the Northumberland Strait (**Figure 4B**). This included rural areas of Nova Scotia such as the Southwest mainland (B0W), Queens (B0T), Hants (B0N) and Northumberland Strait (B0K), as well as Charlottetown (C1A), the capital of PEI. **Supporting Table 4** identifies 24 statistically-significant low incidence FSAs. Exactly 50% of these were located in Ontario within the Greater Toronto Area (GTA).

*Analysis of esophageal squamous cell carcinoma*

Of the 21 cities with the highest incidence of esophageal SCC in Canada (**Supporting Table 5A**), 8 (38%) were found in ON, 6 (29%) in BC, three (14%) in QC, two (9%) in SK, and one (5%) each in AB and MB. In contrast, of the 59 low-incidence cities (**Supporting Table 5B**), 27 (46%) were located in ON, 24 (41%) were located in QC, 3 (5%) in AB, 2 (3%) each in BC and NS, and finally 1 (2%) in NL. Four other FSAs were located nearby in various parts of Vancouver (V7V, V5M) and Burnaby (V5C, V5H). Several additional FSAs were noted on British Columbia's west coast, particularly on the Central Vancouver Island (V0P), Campbell River (V9W), and Parksville (V9P). Finally, the region of Saanich was significantly affected with a cluster in Sidney (V8L), and Victoria (V8X and V8R), seen in **Figure 5A**. Several rural regions of Québec displayed a higher incidence than the national average, namely in Gaspésie (G0E) and Bas-Saint-Laurent-Est (G0K) which border Saint-Basile (E7C), a high incidence FSA in NB (**Figure 5B**). In NS (**Figure 5C**), the north-eastern territory and Cape Breton had higher incidence, with North Victoria County (B0C), Antigonish (B2G), Sydney (B1P, B1V) having significantly higher incidence than the national average. In NL (**Figure 5D**), significant high-incidence FSAs were found in St. John's (A1C) and on the Western Avalon Peninsula (A0B). Low incidence of esophageal SCC was identified in 21 areas (**Supporting Table 6**). Eight FSAs each were found in QC and ON, corresponding to 38% each of low incidence regions in Canada. Three quarters (6/8) of the ON cities were located in the GTA, and all eight of the areas in QC were found in the Greater Montreal Area.

**Reference:**

1. Tsang M, Le M, Ghazawi FM, et al. Multiple myeloma epidemiology and patient geographic distribution in Canada: A population study. Cancer. 2019;125: 2435-2444.

**Supporting Table 1A-B:** Crude incidence of esophageal cancer in Canadian cities. Cities are divided into high incidence, and low incidence compared to the average esophageal cancer incidence rate in Canada. All case numbers are rounded to the nearest 5. All population numbers are rounded to the nearest ten.

***High incidence Canadian cities***

| Cities | Province | Frequency | Population | Crude incidence per million individuals per year | Lower CI(95%) | Upper CI(95%) |
|---|---|---|---|---|---|---|
| **Arnprior** | ON | 10 | 7390 | 71.22 | 34.15 | 130.98 |
| **Nanaimo** | BC | 105 | 76410 | 72.32 | 59.15 | 87.55 |
| **Gravenhurst** | ON | 15 | 10900 | 72.43 | 40.54 | 119.46 |
| **North Saanich** | BC | 15 | 10690 | 73.85 | 41.33 | 121.81 |
| **Peterborough** | ON | 105 | 73820 | 74.86 | 61.23 | 90.63 |
| **Esquimalt** | BC | 25 | 16330 | 80.57 | 52.14 | 118.94 |
| **Moose Jaw** | SK | 50 | 32630 | 80.65 | 59.86 | 106.33 |
| **Thunder Bay** | ON | 10 | 6300 | 83.41 | 40.06 | 153.64 |
| **Brockville** | ON | 35 | 21740 | 84.73 | 59.02 | 117.84 |
| **Elliot Lake** | ON | 20 | 12110 | 86.92 | 53.09 | 134.24 |
| **Algoma** | ON | 10 | 5880 | 89.51 | 42.92 | 164.61 |
| **Kirkland Lake** | ON | 15 | 8730 | 90.43 | 50.61 | 149.15 |
| **Bracebridge** | ON | 25 | 14510 | 90.68 | 58.68 | 133.86 |
| **Summerland** | BC | 20 | 10850 | 97.02 | 59.26 | 149.83 |
| **Powell River** | BC | 25 | 13060 | 100.75 | 65.20 | 148.73 |
| **Trail** | BC | 15 | 7590 | 104.01 | 58.22 | 171.56 |
| **Comox** | BC | 25 | 11120 | 118.33 | 76.57 | 174.67 |
| **Parksville** | BC | 25 | 10690 | 123.09 | 79.65 | 181.70 |
| **Perth** | ON | 15 | 5910 | 133.58 | 74.77 | 220.32 |
| **Sidney** | BC | 30 | 11030 | 143.15 | 96.58 | 204.36 |

### A. *Low incidence Canadian cities*

| Cities | Province | Frequency | Population | Crude incidence per million individuals per year | Lower CI(95%) | Upper CI(95%) |
|---|---|---|---|---|---|---|
| **Dollard-des-Ormeaux** | QC | 5 | 48020 | 5.48 | 1.78 | 12.79 |
| **Grande Prairie** | AB | 5 | 42620 | 6.17 | 2.00 | 14.41 |
| **Blainville** | QC | 5 | 41410 | 6.35 | 2.06 | 14.83 |
| **Brant** | ON | 5 | 32880 | 8.00 | 2.60 | 18.68 |
| **Lakeshore** | QC | 5 | 30670 | 8.58 | 2.79 | 20.02 |
| **Stoney Creek** | ON | 10 | 54000 | 9.75 | 4.67 | 17.92 |
| **Thetford Mines** | QC | 5 | 25710 | 10.24 | 3.32 | 23.89 |
| **Shawinigan** | QC | 10 | 50980 | 10.32 | 4.95 | 18.99 |
| **Sainte-Thérèse** | QC | 5 | 24750 | 10.63 | 3.45 | 24.81 |
| **Centre Wellington** | ON | 5 | 24700 | 10.65 | 3.46 | 24.86 |
| **Wood Buffalo** | AB | 10 | 48440 | 10.87 | 5.21 | 19.98 |
| **Saint-Bruno-de-Montarville** | QC | 5 | 23780 | 11.07 | 3.59 | 25.83 |
| **Saint-Léonard** | QC | 15 | 70470 | 11.20 | 6.27 | 18.48 |
| **Aurora** | ON | 10 | 43960 | 11.97 | 5.74 | 22.02 |
| **Clarence-Rockland** | ON | 5 | 20560 | 12.80 | 4.16 | 29.87 |
| **Chicoutimi** | QC | 15 | 61530 | 12.83 | 7.18 | 21.16 |
| **Whitehorse** | YT | 5 | 20490 | 12.84 | 4.17 | 29.97 |
| **La Prairie** | QC | 5 | 20290 | 12.97 | 4.21 | 30.27 |
| **Lachine** | QC | 10 | 40070 | 13.13 | 6.30 | 24.16 |

**Supporting Table 2A-B:** List of populous Forward Sortation Areas (FSA) in Canada with high and zero age-standardized incidence rates (ASIR) and crude incidence rates of esophageal cancer from 1992 to 2010. All population numbers are rounded to the nearest ten.

**2A: High incidence FSA**

| FSA | Province | Frequency | Population | ASIR per million individuals per year | Lower CI(95%) | Upper CI(95%) |
|---|---|---|---|---|---|---|
| **P5A** | ON | 20 | 12130 | 52.13 | 26.94 | 91.06 |
| **V1R** | BC | 15 | 9950 | 54.06 | 26.18 | 98.76 |
| **V1Y** | BC | 45 | 31970 | 54.93 | 37.91 | 77.00 |
| **V9P** | BC | 40 | 20500 | 57.23 | 35.99 | 86.42 |
| **V0X** | BC | 30 | 19960 | 58.16 | 36.47 | 88.01 |
| **G1M** | QC | 25 | 17760 | 58.86 | 35.89 | 91.03 |
| **P6B** | ON | 35 | 23620 | 60.01 | 39.52 | 87.35 |
| **S4P** | SK | 20 | 12480 | 61.17 | 33.85 | 101.74 |
| **K9J** | ON | 65 | 44730 | 62.54 | 46.87 | 81.77 |
| **V8L** | BC | 45 | 22190 | 63.54 | 41.80 | 92.58 |
| **K6V** | ON | 45 | 28400 | 63.80 | 44.29 | 88.97 |
| **N7A** | ON | 20 | 11680 | 64.43 | 35.47 | 107.54 |
| **V9S** | BC | 25 | 15520 | 67.64 | 41.28 | 104.52 |
| **V6A** | BC | 30 | 16530 | 67.65 | 42.00 | 103.18 |
| **V8A** | BC | 30 | 17930 | 68.36 | 43.48 | 102.32 |
| **V9R** | BC | 35 | 25050 | 69.67 | 48.01 | 97.76 |
| **L7N** | ON | 25 | 12880 | 71.15 | 41.75 | 113.38 |
| **S6H** | SK | 50 | 29770 | 71.97 | 51.58 | 97.74 |
| **K2A** | ON | 30 | 15430 | 74.31 | 46.44 | 112.74 |
| **A1C** | NF | 20 | 14280 | 74.42 | 45.58 | 114.70 |

## 2B: Zero incidence FSA

| FSA | Province | Frequency | Population | Crude incidence per million individuals per year (95% CI) |
|---|---|---|---|---|
| **A0A** | NF | 0 | 107280 | 0 (0-1.81) |
| **G8K** | QC | 0 | 20500 | 0 (0-9.47) |
| **J3X** | QC | 0 | 20100 | 0 (0-9.66) |
| **J5A** | QC | 0 | 23300 | 0 (0-8.33) |
| **L1E** | ON | 0 | 21430 | 0 (0-9.06) |
| **L1P** | ON | 0 | 24100 | 0 (0-8.06) |
| **L3S** | ON | 0 | 46650 | 0 (0-4.16) |
| **L4B** | ON | 0 | 30270 | 0 (0-6.41) |
| **L4S** | ON | 0 | 24060 | 0 (0-8.07) |
| **L5V** | ON | 0 | 39340 | 0 (0-4.94) |
| **L5W** | ON | 0 | 24320 | 0 (0-7.98) |
| **L6C** | ON | 0 | 24670 | 0 (0-7.87) |
| **L6M** | ON | 0 | 39460 | 0 (0-4.92) |
| **L6Z** | ON | 0 | 31680 | 0 (0-6.13) |
| **M3J** | ON | 0 | 24270 | 0 (0-7.99) |
| **M4S** | ON | 0 | 22440 | 0 (0-8.65) |
| **R2N** | MB | 0 | 23630 | 0 (0-8.22) |
| **T6T** | AB | 0 | 24360 | 0 (0-7.97) |

**Supporting Table 3A-B:** Crude incidence of esophageal adenocarcinoma in Canadian cities. Cities are divided into high incidence, and low incidence compared to the average esophageal adenocarcinoma incidence rate in Canada. All case numbers are rounded to the nearest 5. All population numbers are rounded to the nearest thousand.

### A. *High incidence Canadian cities*

| Cities | Province | Frequency | Population | Crude incidence per million individuals per year | Lower CI(95%) | Upper CI(95%) |
|---|---|---|---|---|---|---|
| **Sidney** | BC | 20 | 11000 | 95.43 | 58.29 | 147.39 |
| **Thunder Bay** | ON | 10 | 6000 | 83.41 | 40.00 | 153.39 |
| **Powell River** | BC | 20 | 13000 | 80.60 | 49.23 | 124.48 |
| **Parksville** | BC | 15 | 11000 | 73.85 | 41.33 | 121.81 |
| **Summerland** | BC | 15 | 11000 | 72.76 | 40.72 | 120.01 |
| **Digby** | NS | 10 | 8000 | 64.50 | 30.93 | 118.62 |
| **Kirkland Lake** | ON | 10 | 9000 | 60.29 | 28.91 | 110.87 |
| **Clare** | NS | 10 | 9000 | 59.34 | 28.45 | 109.12 |
| **Bracebridge** | ON | 15 | 15000 | 54.41 | 30.45 | 89.74 |
| **Brighton** | ON | 10 | 10000 | 53.11 | 25.47 | 97.67 |
| **Central Saanich** | BC | 15 | 15000 | 51.23 | 28.67 | 84.50 |
| **Yarmouth** | NS | 10 | 10000 | 50.61 | 24.27 | 93.07 |
| **North Saanich** | BC | 10 | 11000 | 49.23 | 23.61 | 90.54 |
| **Brockville** | ON | 20 | 22000 | 48.42 | 29.58 | 74.78 |
| **Esquimalt** | BC | 15 | 16000 | 48.34 | 27.06 | 79.74 |
| **Gravenhurst** | ON | 10 | 11000 | 48.29 | 23.15 | 88.80 |
| **Ingersoll** | ON | 10 | 11000 | 46.37 | 22.24 | 85.28 |
| **Peterborough** | ON | 65 | 74000 | 46.34 | 35.77 | 59.07 |
| **Saint-Charles-Borromée** | QC | 10 | 12000 | 45.73 | 21.93 | 84.09 |
| **Truro** | NS | 10 | 12000 | 44.60 | 21.39 | 82.03 |
| **Saugeen Shores** | ON | 10 | 12000 | 44.01 | 21.10 | 80.93 |
| **Elliot Lake** | ON | 10 | 12000 | 43.46 | 20.84 | 79.93 |
| **South Stormont** | ON | 10 | 12000 | 43.25 | 20.74 | 79.53 |
| **Nanaimo** | BC | 60 | 76000 | 41.33 | 31.54 | 53.20 |
| **Saint John** | NB | 55 | 70000 | 41.31 | 31.12 | 53.77 |
| **Portage la Prairie** | MB | 10 | 13000 | 40.67 | 19.50 | 74.80 |
| **Moose Jaw** | SK | 25 | 33000 | 40.32 | 26.10 | 59.53 |
| **Woolwich** | ON | 15 | 20000 | 40.32 | 22.57 | 66.50 |
| **Colchester** | ON | 10 | 13000 | 39.69 | 19.03 | 72.99 |
| **Wasaga Beach** | ON | 10 | 13000 | 39.22 | 18.81 | 72.12 |
| **Lincoln** | ON | 15 | 21000 | 37.76 | 21.13 | 62.27 |
| **East Hants** | NS | 15 | 21000 | 37.56 | 21.02 | 61.95 |
| **Cornwall** | ON | 30 | 46000 | 34.07 | 22.99 | 48.64 |
| **Penticton** | BC | 20 | 32000 | 33.22 | 20.29 | 51.30 |
| **Belleville** | ON | 30 | 48000 | 33.16 | 22.38 | 47.34 |
| **Kingston** | ON | 75 | 120000 | 32.89 | 25.87 | 41.23 |
| **Charlottetown** | PEI | 20 | 33000 | 32.01 | 19.56 | 49.44 |
| **Fredericton** | NB | 30 | 50000 | 31.45 | 21.22 | 44.89 |
| **Chilliwack** | BC | 40 | 68000 | 31.16 | 22.26 | 42.43 |

| | | | | | | |
|---|---|---|---|---|---|---|
| **Sault Ste. Marie** | ON | 45 | 76000 | 31.09 | 22.68 | 41.60 |
| **Thunder Bay** | ON | 65 | 110000 | 31.09 | 23.99 | 39.62 |
| **Victoria** | BC | 45 | 76000 | 30.99 | 22.60 | 41.46 |
| **St. Thomas** | ON | 20 | 35000 | 30.37 | 18.55 | 46.90 |
| **Woodstock** | ON | 20 | 35000 | 30.32 | 18.52 | 46.82 |
| **Kamloops** | BC | 45 | 80000 | 29.63 | 21.61 | 39.65 |
| **North Bay** | ON | 30 | 54000 | 29.41 | 19.85 | 41.99 |
| **Langley** | BC | 50 | 91000 | 28.84 | 21.41 | 38.03 |
| **St. Catharines** | ON | 70 | 131000 | 28.15 | 21.95 | 35.57 |
| **New Westminster** | BC | 30 | 57000 | 27.64 | 18.65 | 39.45 |
| **Saanich** | BC | 55 | 106000 | 27.37 | 20.62 | 35.63 |
| **Niagara Falls** | ON | 40 | 80000 | 26.24 | 18.75 | 35.73 |
| **Burlington** | ON | 75 | 157000 | 25.14 | 19.78 | 31.52 |
| **Cape Breton** | NS | 50 | 105000 | 25.04 | 18.59 | 33.01 |

### B. *Low incidence Canadian cities*

| Cities | Province | Frequency | Population | Crude incidence per million individuals per year | Lower CI(95%) | Upper CI(95%) |
|---|---|---|---|---|---|---|
| **Edmonton** | AB | 205 | 706000 | 15.28 | 13.3 | 17.52 |
| **Vancouver** | BC | 140 | 560000 | 13.15 | 11.1 | 15.52 |
| **Laval** | QC | 90 | 361000 | 13.12 | 10.6 | 16.13 |
| **Calgary** | AB | 230 | 933000 | 12.97 | 11.4 | 14.76 |
| **Québec** | QC | 120 | 504000 | 12.53 | 10.4 | 14.99 |
| **Trois-Rivières** | QC | 30 | 129000 | 12.26 | 8.27 | 17.50 |
| **Gatineau** | QC | 55 | 254000 | 11.41 | 8.59 | 14.85 |
| **Terrebonne** | QC | 20 | 101000 | 10.47 | 6.40 | 16.17 |
| **Lévis** | QC | 25 | 134000 | 9.79 | 6.34 | 14.45 |
| **Montréal-Nord** | QC | 15 | 83000 | 9.56 | 5.35 | 15.77 |
| **Ajax** | ON | 15 | 84000 | 9.34 | 5.23 | 15.41 |
| **Pickering** | ON | 15 | 86000 | 9.22 | 5.16 | 15.20 |
| **Montréal** | QC | 285 | 1635000 | 9.17 | 8.14 | 10.30 |
| **Saint-Jean-sur-Richelieu** | QC | 15 | 90000 | 8.78 | 4.91 | 14.48 |
| **Hull** | QC | 10 | 64000 | 8.19 | 3.93 | 15.06 |
| **Toronto** | ON | 395 | 2559000 | 8.12 | 7.34 | 8.97 |
| **Longueil** | QC | 35 | 230000 | 8.00 | 5.57 | 11.12 |
| **East York** | ON | 15 | 108000 | 7.31 | 4.09 | 12.06 |
| **Sainte-Foy** | QC | 10 | 72000 | 7.27 | 3.48 | 13.36 |
| **Mississauga** | ON | 85 | 635000 | 7.05 | 5.63 | 8.71 |
| **Saint-Hubert** | QC | 10 | 76000 | 6.88 | 3.48 | 12.66 |
| **Blainville** | QC | 5 | 41000 | 6.35 | 2.06 | 14.83 |
| **Saint-Eustache** | QC | 5 | 42000 | 6.32 | 2.05 | 14.76 |
| **Brandon** | MB | 5 | 42000 | 6.32 | 2.05 | 14.76 |
| **Grande Prairie** | QC | 5 | 43000 | 6.17 | 2.00 | 14.4 |
| **Brampton** | ON | 45 | 388000 | 6.11 | 4.45 | 8.17 |
| **Drummondville** | QC | 5 | 46000 | 5.75 | 1.87 | 13.43 |
| **Richmond Hill** | ON | 15 | 146000 | 5.43 | 3.04 | 8.95 |
| **Etobicoke** | ON | 35 | 365000 | 5.04 | 3.51 | 7.02 |
| **North York** | ON | 55 | 590000 | 4.91 | 3.70 | 6.39 |
| **Pierrefonds** | QC | 5 | 54000 | 4.87 | 1.58 | 11.36 |
| **Scarborough** | ON | 50 | 559000 | 4.71 | 3.49 | 6.21 |
| **Markham** | ON | 20 | 236000 | 4.45 | 2.72 | 6.88 |
| **Vaughan** | ON | 15 | 210000 | 3.75 | 2.10 | 6.19 |
| **Saguenay** | QC | 10 | 144000 | 3.65 | 1.72 | 6.58 |
| **York** | ON | 10 | 147000 | 3.58 | 1.72 | 6.58 |
| **Saint-Laurent** | QC | 5 | 76000 | 3.47 | 1.13 | 8.10 |

**Supporting Table 4.** List of populous Forward Sortation Areas (FSA) in Canada with zero incidence (crude) of esophageal adenocarcinoma from 1992 to 2010. All population numbers are rounded to the nearest thousand.

| FSA | Province | Average Population | Crude incidence per million individuals per year (95% CI) |
|---|---|---|---|
| **A0A** | NF | 54000 | 0 (0-3.62) |
| **A0N** | NF | 15000 | 0 (0-13.07) |
| **E0L** | NB | 16000 | 0 (0-11.78) |
| **G7B** | QC | 19000 | 0 (0-10.43) |
| **G7G** | QC | 17000 | 0 (0-11.37) |
| **H4K** | QC | 11000 | 0 (0-17.46) |
| **H7H** | QC | 11000 | 0 (0-17.59) |
| **J5J** | QC | 11000 | 0 (0-17.86) |
| **J5K** | QC | 12000 | 0 (0-16.71) |
| **L1P** | ON | 12000 | 0 (0-16.11) |
| **L3S** | ON | 47000 | 0 (0-4.16) |
| **L4B** | ON | 30000 | 0 (0-6.41) |
| **L4K** | ON | 13000 | 0 (0-14.54) |
| **L4S** | ON | 24000 | 0 (0-8.07) |
| **L5W** | ON | 12000 | 0 (0-15.97) |
| **L6E** | ON | 15000 | 0 (0-12.60) |
| **L6Z** | ON | 32000 | 0 (0-6.13) |
| **M3J** | ON | 24000 | 0 (0-8.65) |
| **M3L** | ON | 17000 | 0 (0-11.20) |
| **M4S** | ON | 22000 | 0 (0-8.65) |
| **M4W** | ON | 12000 | 0 (0-15.61) |
| **R2N** | MB | 24000 | 0 (0-8.22) |
| **R2R** | MB | 19000 | 0 (0-10.23) |
| **T6T** | AB | 12000 | 0 (0-15.94) |

**Supporting Table 5A-B:** Crude incidence of esophageal squamous cell carcinoma in Canadian cities. Cities are divided into high incidence, and low incidence compared to the average esophageal squamous cell carcinoma incidence rate in Canada. All case numbers are rounded to the nearest 5. All population numbers are rounded to the nearest ten.

A. ***High incidence Canadian cities***

| City | Province | Cases | Population | Crude incidence per million individuals per year | Lower 95% CI | Upper 95% CI |
|---|---|---|---|---|---|---|
| **Peterborough** | ON | 40 | 73820 | 28.52 | 20.37 | 38.84 |
| **North Cowichan** | BC | 15 | 26950 | 29.29 | 16.38 | 48.32 |
| **Nanaimo** | BC | 45 | 76410 | 31.00 | 22.61 | 41.48 |
| **Camrose** | AB | 10 | 15370 | 34.24 | 16.39 | 62.98 |
| **Gaspé** | QC | 10 | 15360 | 34.27 | 16.40 | 63.02 |
| **Sault Ste. Marie** | ON | 50 | 76180 | 34.54 | 25.64 | 45.54 |
| **Stratford** | ON | 20 | 30020 | 35.06 | 21.41 | 54.16 |
| **Fort Saskatchewan** | SK | 10 | 14880 | 35.37 | 16.93 | 65.05 |
| **Bracebridge** | ON | 10 | 14510 | 36.27 | 17.37 | 66.71 |
| **Brockville** | ON | 15 | 21740 | 36.31 | 20.31 | 59.90 |
| **Moose Jaw** | SK | 25 | 32630 | 40.32 | 26.09 | 59.53 |
| **Cowansville** | QC | 10 | 12190 | 43.18 | 20.67 | 79.41 |
| **Elliot Lake** | ON | 10 | 12110 | 43.46 | 20.81 | 79.93 |
| **Sidney** | BC | 10 | 11030 | 47.72 | 22.84 | 87.76 |
| **Parksville** | BC | 10 | 10690 | 49.23 | 23.57 | 90.55 |
| **Saint-Calixte** | QC | 5 | 5300 | 49.65 | 16.00 | 115.87 |
| **Carleton Place** | ON | 10 | 9210 | 57.15 | 27.36 | 105.10 |
| **Dauphin** | MB | 10 | 8130 | 64.74 | 30.99 | 119.06 |
| **Trail** | BC | 10 | 7590 | 69.34 | 33.20 | 127.53 |
| **Perth** | ON | 10 | 5910 | 89.06 | 42.63 | 163.79 |
| **Comox** | BC | 20 | 11120 | 94.66 | 57.80 | 146.20 |

### *B. Low incidence Canadian cities*

| City | Provinces | Cases | Population | Crude incidence per million individuals per year | Lower 95% CI | Upper 95% CI |
|---|---|---|---|---|---|---|
| **Saguenay** | QC | 10 | 144220 | 3.65 | 1.75 | 6.71 |
| **Brossard** | QC | 5 | 65480 | 4.02 | 1.30 | 9.38 |
| **Jonquière** | QC | 5 | 55670 | 4.73 | 1.52 | 11.03 |
| **Port Coquitlam** | BC | 5 | 51740 | 5.09 | 1.64 | 11.87 |
| **Milton** | ON | 5 | 50460 | 5.22 | 1.68 | 12.17 |
| **Dollard-des-Ormeaux** | QC | 5 | 48020 | 5.48 | 1.77 | 12.79 |
| **Drummondville** | QC | 5 | 45740 | 5.75 | 1.85 | 13.43 |
| **Waterloo** | ON | 10 | 90190 | 5.84 | 2.79 | 10.73 |
| **Haldimand** | ON | 5 | 43960 | 5.99 | 1.93 | 13.97 |
| **Châteauguay** | QC | 5 | 42780 | 6.15 | 1.98 | 14.36 |
| **Gatineau** | QC | 30 | 253740 | 6.22 | 4.20 | 8.88 |
| **Ajax** | ON | 10 | 84490 | 6.23 | 2.98 | 11.46 |
| **Vaughan** | ON | 25 | 210430 | 6.25 | 4.05 | 9.23 |
| **Saint-Eustache** | QC | 5 | 41610 | 6.32 | 2.04 | 14.76 |
| **Victoriaville** | QC | 5 | 40240 | 6.54 | 2.11 | 15.26 |
| **Strathcona County** | AB | 10 | 77790 | 6.77 | 3.24 | 12.44 |
| **Saint-Hyacinthe** | QC | 5 | 38870 | 6.77 | 2.18 | 15.80 |
| **Clarington** | ON | 10 | 73200 | 7.19 | 3.44 | 13.22 |
| **Etobicoke** | ON | 50 | 365140 | 7.21 | 5.35 | 9.50 |
| **Beauport** | QC | 10 | 72870 | 7.22 | 3.46 | 13.28 |
| **Scarborough** | ON | 80 | 559000 | 7.53 | 5.97 | 9.37 |
| **Newmarket** | ON | 10 | 69300 | 7.59 | 3.64 | 13.97 |
| **Markham** | ON | 35 | 236320 | 7.79 | 5.43 | 10.84 |
| **Lévis** | QC | 20 | 134390 | 7.83 | 4.78 | 12.10 |
| **Terrebonne** | QC | 15 | 100510 | 7.85 | 4.39 | 12.96 |
| **Mascouche** | QC | 5 | 33480 | 7.86 | 2.53 | 18.34 |
| **Cap-de-la-Madeleine** | QC | 5 | 32990 | 7.98 | 2.57 | 18.62 |
| **Whitby** | ON | 15 | 98600 | 8.01 | 4.48 | 13.21 |
| **North York** | ON | 90 | 590000 | 8.03 | 6.46 | 9.87 |
| **Trois-Rivières** | QC | 20 | 128820 | 8.17 | 4.99 | 12.62 |
| **Greater Sudbury** | ON | 25 | 159670 | 8.24 | 5.33 | 12.17 |
| **Mississauga** | ON | 100 | 634840 | 8.29 | 6.75 | 10.08 |
| **Norfolk** | ON | 10 | 61780 | 8.52 | 4.08 | 15.67 |
| **Lakeshore** | ON | 5 | 30670 | 8.58 | 2.77 | 20.02 |
| **Sherbrooke** | QC | 25 | 151010 | 8.71 | 5.64 | 12.86 |
| **Saint-Jean-sur-Richelieu** | QC | 15 | 89940 | 8.78 | 4.91 | 14.48 |
| **Longueuil** | QC | 40 | 230370 | 9.14 | 6.53 | 12.44 |
| **Pickering** | ON | 15 | 85670 | 9.22 | 5.15 | 15.20 |
| **St. Albert** | ON | 10 | 54800 | 9.60 | 4.60 | 17.66 |
| **Repentigny** | QC | 10 | 54190 | 9.71 | 4.65 | 17.86 |
| **East York** | ON | 20 | 108000 | 9.75 | 5.95 | 15.05 |
| **Stoney Creek** | ON | 10 | 54000 | 9.75 | 4.67 | 17.93 |

| | | | | | | |
|---|---|---|---|---|---|---|
| **Calgary** | AB | 175 | 933150 | 9.87 | 8.46 | 11.45 |
| **Toronto** | ON | 485 | 2559000 | 9.98 | 9.11 | 10.90 |
| **Boisbriand** | QC | 5 | 26310 | 10.00 | 3.22 | 23.34 |
| **Port Moody** | BC | 5 | 26290 | 10.01 | 3.23 | 23.36 |
| **Caledon** | ON | 10 | 51750 | 10.17 | 4.87 | 18.70 |
| **Brampton** | ON | 75 | 387850 | 10.18 | 8.00 | 12.76 |
| **Halton Hills** | ON | 10 | 51220 | 10.28 | 4.92 | 18.90 |
| **Saint-Hubert** | QC | 15 | 76480 | 10.32 | 5.77 | 17.03 |
| **Shawinigan** | QC | 10 | 50980 | 10.32 | 4.94 | 18.99 |
| **Lunenburg** | NS | 5 | 25450 | 10.34 | 3.33 | 24.13 |
| **Halifax** | NS | 75 | 381000 | 10.36 | 8.15 | 12.99 |
| **Saint-Laurent** | QC | 15 | 75820 | 10.41 | 5.82 | 17.17 |
| **Red Deer** | AB | 15 | 75380 | 10.47 | 5.86 | 17.28 |
| **Mount Pearl** | NF | 5 | 24860 | 10.59 | 3.41 | 24.70 |
| **York** | ON | 30 | 147000 | 10.74 | 7.25 | 15.33 |
| **Richmond Hill** | ON | 30 | 145500 | 10.85 | 7.32 | 15.49 |
| **Ottawa** | ON | 175 | 848000 | 10.86 | 9.31 | 12.60 |

**Supporting Table 6.** List of populous Forward Sortation Areas (FSA) in Canada with zero incidence (crude) of esophageal squamous cell carcinoma from 1992 to 2010. All population numbers are rounded to the nearest ten.

| FSA | Description | Average population | Crude incidence per million individuals per year (95% CI) |
|---|---|---|---|
| **A0A** | NF | 53640 | 0 (0-3.60) |
| **G1B** | QC | 14550 | 0 (0-13.27) |
| **H4J** | QC | 17380 | 0 (0-11.11) |
| **H7T** | QC | 16580 | 0 (0-11.64) |
| **J3X** | QC | 20100 | 0 (0-9.60) |
| **J4X** | QC | 16130 | 0 (0-11.97) |
| **J5A** | QC | 23300 | 0 (0-8.29) |
| **J5C** | QC | 16490 | 0 (0-11.71) |
| **J5M** | QC | 15800 | 0 (0-12.22) |
| **L1E** | ON | 21430 | 0 (0-9.01) |
| **L1M** | ON | 13820 | 0 (0-13.97) |
| **L2S** | ON | 16920 | 0 (0-11.41) |
| **L5V** | ON | 39340 | 0 (0-4.91) |
| **L6C** | ON | 24670 | 0 (0-7.83) |
| **L6M** | ON | 39460 | 0 (0-4.90) |
| **M5V** | ON | 15340 | 0 (0-12.59) |
| **N2T** | ON | 17320 | 0 (0-11.15) |
| **S4V** | SK | 19530 | 0 (0-9.89) |
| **T9K** | AB | 17700 | 0 (0-10.91) |
| **V9Z** | BC | 15610 | 0 (0-12.37) |
| **X0A** | NU | 15050 | 0 (0-12.83) |

**Supporting Table 7A-B:** Prevalence of different subtypes of esophageal cancer in Canada between 1992 and 2010.

## A. Esophageal adenocarcinoma

| Esophageal adenocarcinoma subtypes | ICD-O-3 Code | No. of patients | Percentage of total |
|---|---|---|---|
| **Adenocarcinoma** | 8140 | 10345 | 96.91 |
| **Adenosquamous carcinoma** | 8560 | 190 | 1.78 |
| **Undifferentiated carcinoma** | 8020 | 125 | 1.17 |
| **Adenoid cystic carcinoma** | 8200 | 5 | 0.05 |
| **Mucoepidermoid carcinoma** | 8430 | 10 | 0.09 |
| **Overall esophageal adenocarcinoma** | - | 10675 | 100 |

## B. Esophageal squamous cell carcinoma

| Esophageal squamous cell carcinoma subtypes | ICD-O-3 Code | No. of patients[a] | Percentage of total |
|---|---|---|---|
| **Squamous cell carcinoma** | 8070 | 9030 | 99.23 |
| **Verrucous carcinoma** | 8051 | 10 | 0.11 |
| **Basaloid squamous cell carcinoma** | 8083 | 60 | 0.66 |
| **Overall esophageal squamous cell carcinoma** | - | 9100 | 100 |

## Supporting Figure Legend

**Supporting Figure 1: Esophageal cancer incidence trends by Canadian city (A) and FSA (B).**

Geographic maps illustrate incidence rates of esophageal cancer (cases per million individuals per year) relative to the national average based on the Canadian Cancer Registry/Quebec Cancer Registry databases.

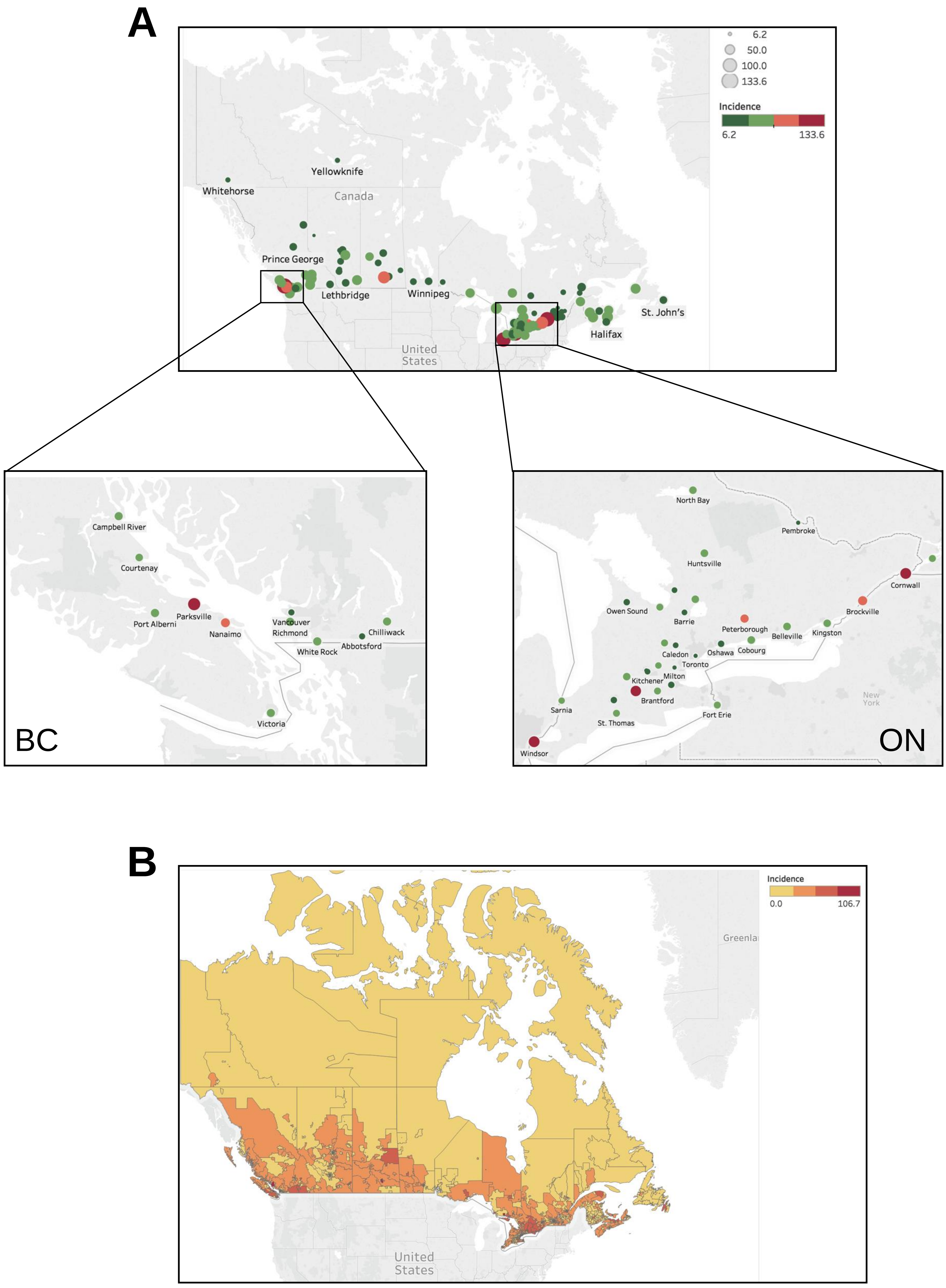


**Supporting Figure 1.**
